# *Ficus lindsayana* Leaf Extract Protects C2C12 Mouse Myoblasts Against the Suppressive Effects of Bisphenol-A on Myogenic Differentiation

**DOI:** 10.3390/ijms26020476

**Published:** 2025-01-08

**Authors:** Pornsiri Pitchakarn, Jirarat Karinchai, Pensiri Buacheen, Arisa Imsumran, Ariyaphong Wongnoppavich, Kongsak Boonyapranai, Sakaewan Ounjaijean

**Affiliations:** 1 Department of Biochemistry, Faculty of Medicine, Chiang Mai University, Chiang Mai 50200, Thailand; pornsiri.p@cmu.ac.th (P.P.); jirarat.ka@cmu.ac.th (J.K.); pensiri.bua@cmu.ac.th (P.B.); arisa.bonness@cmu.ac.th (A.I.); ariyaphong.w@cmu.ac.th (A.W.); 2 Research Center for Non-Infectious Diseases and Environmental Health Sciences, Research Institute for Health Sciences, Chiang Mai University, Chiang Mai 50200, Thailand; kongsak.b@cmu.ac.th; 3 School of Health Sciences Research, Research Institute for Health Sciences, Chiang Mai University, Chiang Mai 50200, Thailand

**Keywords:** pollutants, endocrine-disrupting chemicals, myogenesis, muscle, *Ficus lindsayana*

## Abstract

Recently, toxicological and epidemiological research has provided strong support for the unfavorable effects of bisphenol-A (BPA, 2,2′-bis(4-hydroxyphenyl) propane) on myogenesis and its underlying mechanisms. Researchers have therefore been looking for new strategies to prevent or mitigate these injurious effects of BPA on the human body. It has been found that plant extracts may act as potential therapeutic agents or functional foods, preventing human diseases caused by BPA. We previously reported that *Ficus lindsayana* (FL) extract exhibits anti-inflammation activity in macrophages via suppressing the expression of inflammation-related molecules and anti-insulin resistance in inflammation-treated adipocytes. In this study, we investigated whether *Ficus lindsayana* leaf extract (FLLE) protects C2C12 mouse myoblasts against the suppressive effects of BPA on myogenic differentiation. The viability of BPA-stimulated C2C12 myoblasts was significantly increased when co-treated with FLLE (200 µg/mL), suggesting that the extract may lessen the inhibitory effects of BPA on cell division. We also found that FLLE significantly increased neo-myotube formation by inducing the fusion of myoblasts into multinucleated myotubes when compared to the BPA-treated control cells, without impacting cell viability. In addition, the levels of myogenin and myocyte enhancer factor 2A (MEF2A), which are crucial markers and regulators of myogenesis, were markedly increased by the addition of FLLE (50 µg/mL) to the BPA-treated C2C12 cells. This finding suggests that FLLE effectively improved myogenic differentiation in BPA-exposed myoblasts. FLLE treatment (50 µg/mL) significantly raised total Akt protein levels in the BPA-treated C2C12 cells, enhancing protein phosphorylation. In addition, FLLE (50 µg/mL) obviously increased the phosphorylation levels of p70S6K and 4E-BP1, key downstream targets of the Akt/mTOR signaling cascade, by elevating total p70S6K and 4E-BP1 levels. These results suggest that FLLE diminishes the decline in myogenic differentiation induced by BPA via the regulation of the myocyte differentiation-related signaling pathway. The information obtained from this study demonstrates the health benefits of this plant, which warrants further investigation as an alternative medicine, functional ingredient, or food supplement that can prevent the negative health effects of BPA or other toxicants.

## 1. Introduction

Skeletal muscle is the most common tissue found in vertebrates and is essential for body mobility and metabolism. The maintenance of muscle mass is critical for the prevention of metabolic syndrome and age-related muscle loss, which impair quality of life [1]. Skeletal myoblast differentiation is a highly complex process involving myoblast growth, cell cycle withdrawal, the expression of muscle-specific genes, and fusion into multinucleated myofibers [2]. During embryonic development, the mesoderm gives rise to skeletal muscle tissue, the precursor cells for muscle, under the influence of myogenic regulatory factors (MRFs). Key MRFs involved in this process include MyoD, myogenin, myogenic factor 5 (myf-5), and myogenic regulatory factor 4 (MRF4), all of which are crucial for muscle development [3]. Myogenesis consists of two essential stages. The first stage involves the proliferation of myoblasts, which increases the number of muscle cells and is regulated by the transcription factors MyoD and Myf-5. The second stage is characterized by the differentiation of myoblasts, during which they produce myosin heavy chains (MHC), the main structural protein found in muscle tissue. Myogenin and MRF4 are crucial transcription factors involved in myogenesis and govern the fusion and maturation of these myocytes into multinucleated myofibers [4,5]. MEF2A, a transcription factor that binds to specific promoters of myogenic genes, has been identified as a positive regulator of skeletal muscle myoblast proliferation and differentiation. It was found that MEF2A cooperates with other MRFs to activate muscle-specific gene expression, effectively driving the myogenic differentiation program [6,7,8,9]. Some mesodermal cells produce satellite cells during myogenic development, which are necessary for muscle growth and regeneration after birth. Satellite cells are found between muscle fibers in adult muscle. Satellite cells are activated and MRFs are produced in response to muscle injury, resulting in muscle regeneration, which involves satellite cell proliferation and differentiation, as well as promoting the survival of satellite cells and their integration into functioning myofibers [10,11].

Impairments in myogenesis and muscle regeneration lead to deformities or atrophy of skeletal muscle, a characteristic sign of muscle aging that impacts body movement [12]. Several exogenous and endogenous factors influence the rate of adult myogenesis. Positive influences include exercise, growth factors, hormones, and nutrients [13,14,15,16,17]. Aging, illnesses, and pollutants from the environment are all unfavorable factors that inhibit myogenesis [18,19,20]. Recent studies have discovered the presence of estrogen receptor (ER) α and ERβ in myoblasts and skeletal muscle tissues [21,22]. This suggests that estrogens, along with estrogenic endocrine-disrupting chemicals (EDCs)—a diverse range of compounds that can either mimic or block the effects of natural estrogens [23]—interact with skeletal muscle via these ER isoforms [24]. Furthermore, recent toxicological and epidemiological research has strongly indicated that bisphenol-A (BPA or 2,2′-bis(4-hydroxyphenyl) propane) adversely affects myogenesis and elucidated the mechanisms behind this impact [25,26]. A previous study demonstrated that BPA causes myocardial fibrosis by stimulating the ERK1/2 pathway [27]. Furthermore, the toxicity of BPA has been identified to occur via the activation of intracellular signaling pathways related to ERs [28]. Studies in C2C12 mouse myoblasts reported that BPA significantly diminished myoblast proliferation and differentiation through downregulation of the expression of the myogenic regulatory factor gene via the suppression of AKT, JNK, and P65 NF-κB activity, leading to apoptotic cell death and/or muscle atrophy [3,29].

BPA, a xeno-estrogen polymeric component, is widely used worldwide in the manufacture of polycarbonate and epoxy resin. It has a short half-life and is released after exposure to elevated temperature. People are persistently exposed to low doses of this chemical, primarily through modern fast-food, processed/packaged food diets, water bottles, dust, dental sealants, thermal paper, and particulate matter (PM2.5) [30,31,32,33]. BPA and its metabolites can be found in human body fluids such as blood, urine, saliva, amniotic fluid, and breast milk [34,35,36,37]. Moreover, BPA has been detected in the liver, muscle, and brain tissues of the offspring of Balb-C mice exposed to the compound [38]. This indicates that exposure to BPA can occur frequently and BPA can accumulate in the body, which causes adverse effects on health [36,37]. Avoidance of exposure to BPA in daily life is hard due to its widespread distribution in our food chain and the inadequate labeling of foods packaged in BPA-containing substances [39]. A previous study in teenagers found that detailed information pertinent to avoiding BPA exposure in the long term via self-directed modification of diet in a ‘real-world’ setting is limited. Furthermore, the participants were hesitant to make such long-term lifestyle changes due to the food restrictions required and the implications for daily living [40]. Recently, researchers have been looking for new strategies to prevent or mitigate the detrimental effects of BPA on the human body. It was found, both in vitro and in vivo, that various natural and synthetic antioxidants could overcome the toxicity of BPA [41]. *Asparagus officinalis* extract could increase the total antioxidative capability, improving the function and structure of the liver and kidney tissues of male rats that were orally administered with BPA [42]. Banerjee, O. et al. reported that *Centella asiatica* (CA) ethanol extract inhibits BPA-induced pancreatic islet toxicity in male Swiss albino mice [43]. Our previous study found that *Anoectochilus burmannicus* extract, which has antioxidant and anti-inflammation abilities, suppresses BPA-stimulated adipocyte formation by downregulating the expression of adipogenic genes, leading to a decrease in lipid accumulation in adipocytes [44]. These findings suggest that plant extracts may act as potential therapeutic agents or functional foods preventing human diseases caused by BPA.

For centuries, approximately 800 species within the Ficus genus have been utilized as food and for medicinal purposes in both Ayurvedic and traditional Chinese medicine [45]. These species are prevalent in tropical and subtropical regions of India, China, Sri Lanka, Australia, and Myanmar. In traditional healing practices, various parts of Ficus plants—such as bark, fruits, latex, leaves, roots, and twigs—have been used to treat a range of ailments, including diarrhea, digestive issues, cancer, inflammation, and diabetes [46,47]. Additionally, Ficus species have demonstrated antioxidant and antimicrobial properties [48,49], and *Ficus* spp. have been reported to have significant therapeutic effects in chronic diseases [47]. Flavonoids found in *Ficus* sp. extracts have been shown to lower blood glucose levels and also have anti-cancer, antioxidant, antihistaminic, and anti-inflammatory effects [48,50,51].

Another *Ficus* spp. which has the potential to be employed as an alternative medicine or a functional food is *Ficus lindsayana* (FL) Beentje, formerly called *Ficus dubia* Wall. ex King [52] (the plant name was checked with the WFO (http://www.worldfloraonline.org/taxon/wfo-1000027124, accessed on 25 July 2024)). This tree is native to the tropical evergreen rainforests of Southern Thailand, Malaysia, and Brunei [53], where it can grow up to 30–35 m tall and features aerial roots that descend from its branches or trunk. Notably, it produces a distinctive red latex, which locals believe to correlate with health benefits similar to those of dragon’s blood—a traditional remedy sourced from various plants including *Croton* spp., *Daemonorops* spp., *Dracaena* spp. and *Pterocarpus* spp. [54]. Previous studies have identified several biological activities associated with different parts of FL. Extracts from its stems and twigs have demonstrated anti-HIV and antibacterial properties [55]. Additionally, both root and latex extracts of FL show significant antioxidant activity, likely due to the high levels of phenolics and flavonoids [56]. Ethanolic extract from the roots has been found to possess strong antidiabetic effects by inhibiting α-glucosidase, a key target in diabetes treatment, as well as having anti-cancer properties against lung and ovarian cancer cell lines, without causing genomic damage [56]. Furthermore, latex extracts have shown promise in preventing and treating colorectal cancer through the modulation of inflammatory pathways [57,58]. The root and latex extracts of FL showed the ability to induce adipogenesis, which might prevent the formation of hypertrophic adipocytes, contributing to a decline in inflammation, dysfunction, and insulin resistance [59]. Moreover, the extracts could exhibit anti-inflammatory activity in macrophages via the suppression of the expression of inflammation-related molecules [59].

The present study aims to determine whether *Ficus lindsayana* leaf extract protects C2C12 mouse myoblasts against the suppressive effects of BPA on myogenic differentiation. The findings of this study will provide evidence of the plant’s health benefits, allowing for its further development as an alternative medicine, functional ingredient, or dietary supplement aimed at mitigating the negative health effects associated with BPA and other toxic substances.

## 2. Results

### 2.1. Phytochemical Compounds in and FLLE Obtained Using HPLC

**Figure 1:**
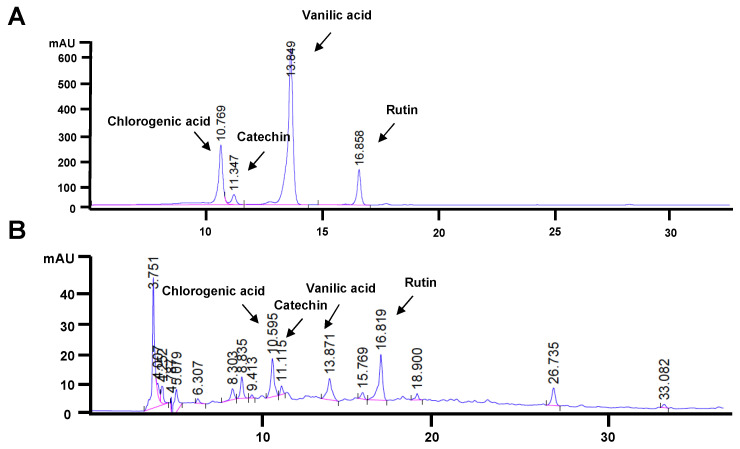
HPLC chromatogram of the standard mixture containing chlorogenic acid, catechin, vanillic acid, and rutin (**A**). Phytochemical profile of FLLE analyzed by HPLC (**B**).

From the HPLC chromatograms, chlorogenic acid, catechin, vanillic acid, and rutin were detectable in FLLE (Figure 1B) when compared with the HPLC fingerprint of the standard compounds (Figure 1A). The chlorogenic acid, catechin, vanillic acid, and rutin contents were 3.09 ± 0.076, 1.08 ± 0.053, 0.97 ± 0.020, and 5.51 ± 0.155 mg/g extracts, respectively, as shown in Table 1. The HPLC fingerprint of all standards used in this study is shown in Supplementary Figure S1A,B. The standard curves of chlorogenic acid, rutin, vanillic acid, and catechin that were detectable in FLLE are shown in Supplementary Figure S1C–F.

### 2.2. Effect of FLLE on the Viability of C2C12 Myoblasts

**Figure 2:**
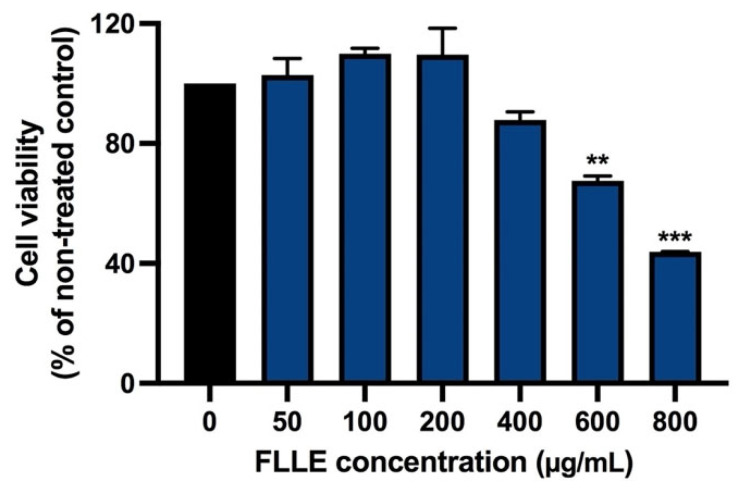
Effect of FLLE on the viability of C2C12 myoblasts. The cells were treated with various concentrations of the extracts (0–800 µg/mL) for 48 h. Cell viability was determined via MTT assay. Each value represents mean ± SD (n = 3) ** *p* < 0.01 and *** *p* < 0.001 vs. non-treated control.

As shown in Figure 2, FLLE, at doses up to 200 µg/mL, was not cytotoxic in non-differentiation C2C12 myoblasts (48 h). The extract at 400 µg/mL slightly decreased the cell viability of C2C12 myoblasts, while the cell number was significantly reduced when the cells were treated with 600–800 µg/mL of the extract. The inhibitory concentrations of 20 and 50 (IC20 and IC50) of the extract were approximately 400 and 700 µg/mL, respectively.

These results are consistent with our previous study, which reported that latex and root extracts of FL were not cytotoxic to various kinds of normal cells [59]. To ensure FLLE’s effects on the myogenesis of BPA-treated C2C12, cytotoxicity was measured in the cells cotreated with BPA and FLLE (0–200 µg/mL).

### 2.3. Effect of FLLE on the Viability of BPA-Treated C2C12 Myoblasts

**Figure 3:**
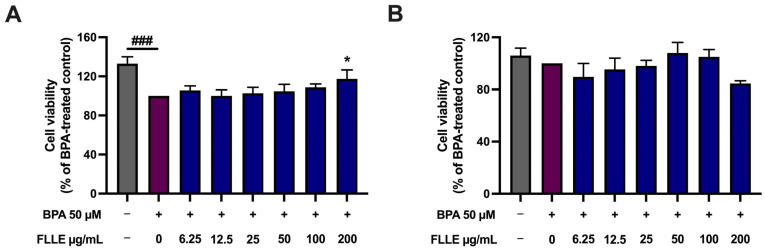
Effect of BPA and FLLE co-treatment on the viability of C2C12 myoblasts under non-differentiated (**A**) and differentiated conditions (**B**). (**A**) The nondifferentiated C2C12 myoblasts were treated with various concentrations of the extracts (0–200 µg/mL) in the presence or absence of 50 µM BPA for 72 h. (**B**) The cells were treated with the extracts (0–200 µg/mL) in the presence or absence of 50 µM BPA during differentiation for 6 days. At the indicated time, the cell viability was determined via MTT assay. Each value represents mean ± SD (n = 3) * *p* < 0.05 vs. BPA-treated control, ^###^ *p* < 0.001 vs. non-treated control.

As shown in Figure 3A, BPA (50 µM) significantly decreased the cell viability of C2C12 myoblasts in the non-differentiated condition, suggesting the inhibition of cell proliferation. This result is similar to that of a previous report, which shows an inhibitory effect of BPA on the cell cycle of C2C12 myoblasts [3]. Interestingly, the viability of the BPA-stimulated C2C12 myoblasts was significantly increased when the cells were co-treated with 200 µg/mL FLLE, suggesting that the extract may reduce the inhibitory effect of BPA on cell division. In addition, our preliminary study found that treatment with 50 µM of BPA during differentiation for 6 days significantly decreased the fusion index of C2C12 (Supplementary Figure S2), while a high concentration of PBA (100 µM) caused marked cell death when observed under microscope. Therefore, 50 µM of BPA was used in all the following experiments.

It was found that BPA (50 µM) treatment during the myogenesis of C2C12 myoblasts, with or without FLLE, did not have any significant impact on cell death (Figure 3B). Normally, when the cells were cultured in the differentiation medium, the proliferation of the cells was inhibited while their transformation to myotubes was stimulated. These data confirm that BPA may have an inhibitory effect on the proliferation of the myoblasts rather than inducing cell death during differentiation. As FLLE slightly affected the cell viability of C2C12 during the 6-day treatment (during differentiation), a concentration of FLLE of up to 50 µg/mL was used to determine its effect on the myogenesis of BPA-treated C2C12 myoblasts.

### 2.4. Effect of FLLE on the Myogenesis of BPA-Treated C2C12 Myoblasts

**Figure 4:**
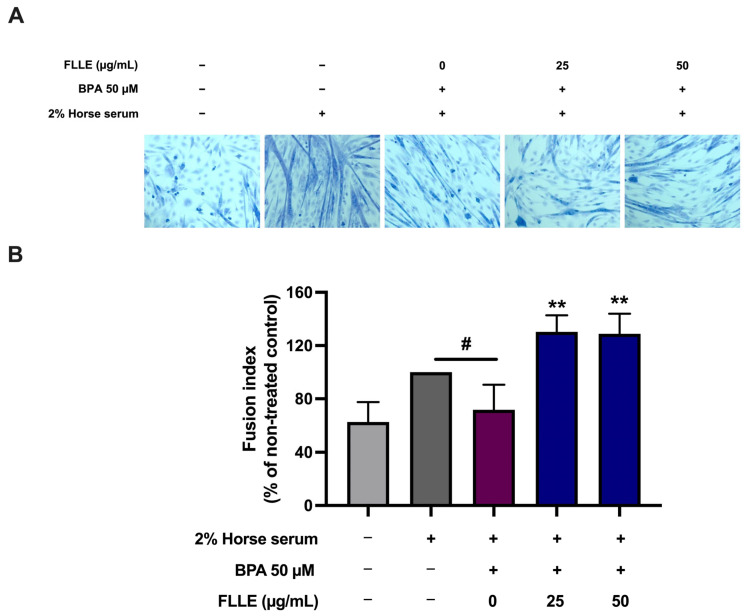
Effects of FLLE on the myogenesis of BPA-treated C2C12 myoblasts. The cells were treated with the extracts (0–50 µg/mL) in the presence of 50 µM BPA during differentiation for 6 days. (**A**) Morphological changes in C2C12 cells under microscopic observation on the sixth day of differentiation. (**B**) The effects of FLLE on myogenic differentiation, determined by measuring the fraction of nuclei incorporated into myotubes on the sixth day of myogenic differentiation. Each value represents mean ± SD (n = 3) ** *p* < 0.01 vs. BPA-treated control, ^#^ *p* < 0.05 vs. non-treated control.

To investigate the ability of FLLE to suppress BPA-inhibited muscle differentiation, C2C12 cells cultured in a differentiation medium were treated with FLLE (25, 50 µg/mL) in the presence of BPA for 6 days. Subsequently, we examined the effects of FLLE on the BPA-induced morphological changes in C2C12 cells that were associated with the differentiation process (Figure 4A). Microscopic observation on the sixth day of differentiation revealed that BPA suppressed the differentiation of C2C12 myoblasts. The cells showed a typical triangular morphology and their shape did not change into a new elongated shape. However, morphological changes in C2C12 cells representing myotube formation were observed following co-treatment with FLLE (25, 50 µg/mL) (Figure 4A). These findings indicate that FLLE increased the myogenic differentiation of C2C12 cells. Next, the effect of FLLE on myogenic differentiation was examined through an assessment of the fraction of nuclei integrated into myotubes on the sixth day of differentiation. Figure 4B demonstrated that the fusion index of the BPA-treated group (+BPA) significantly decreased when compared to the differentiation control group (−BPA). Interestingly, the fusion index was significantly increased following FLLE treatment (25, 50 µg/mL) in BPA-treated C2C12 myoblasts.

**Figure 5:**
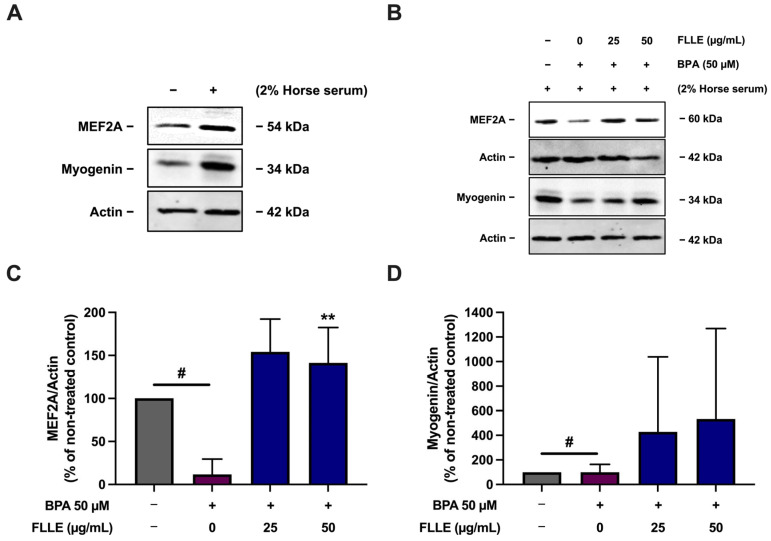
Effect of FLLE on the protein level of MEF2A and myogenin myogenesis markers in BPA-treated C2C12 myoblasts. The cells were treated with the extracts (0–50 µg/mL) in the absence or presence of 50 µM BPA during differentiation for 6 days. After the treatment, the protein samples were collected and myogenin and MEF2A levels were determined via Western blotting (normalized with β-actin level). (**A**) The expression of MEF2A and myogenin in non-differentiated myoblasts compared to non-treated control myocytes. (**B**) A representative result of three independent experiments. (**C**,**D**) Relative band density of MEF2A (**C**) and myogenin (**D**) normalized with β-actin level. Each value in represents mean ± SD (n = 3) ** *p* < 0.01 vs. BPA-treated control, ^#^ *p* < 0.05 vs. non-treated control.

To confirm that FLLE successfully induced myogenesis in BPA-treated cells, the level of myogenic marker proteins, including myogenin and MEF2A, was then measured. As shown in Figure 5, BPA dramatically diminished the expression of proteins. Interestingly, FLLE (50 µg/mL) significantly stimulated the protein levels of MEF2A in the BPA-treated cells, while the expression levels of myogenin were obviously but not significantly increased through this treatment. As shown in Supplementary Figure S3, the variation in BPA’s effect on myogenin protein level, determined via tree-time independent experiments, resulted in a high range of standard deviation (SD), contributing to a non-significant data set. The results obtained from three time-independent experiments revealed that FLLE could effectively increase the expression of myogenin in the BPA-treated cells. Taken together, these results indicate that FLLE protects C2C12 mouse myoblasts against the suppressive effects of BPA on myogenic differentiation.

### 2.5. Effects of FLLE on the Akt/p70S6K/4EBP Pathway in C2C12 Myotubes

**Figure 6:**
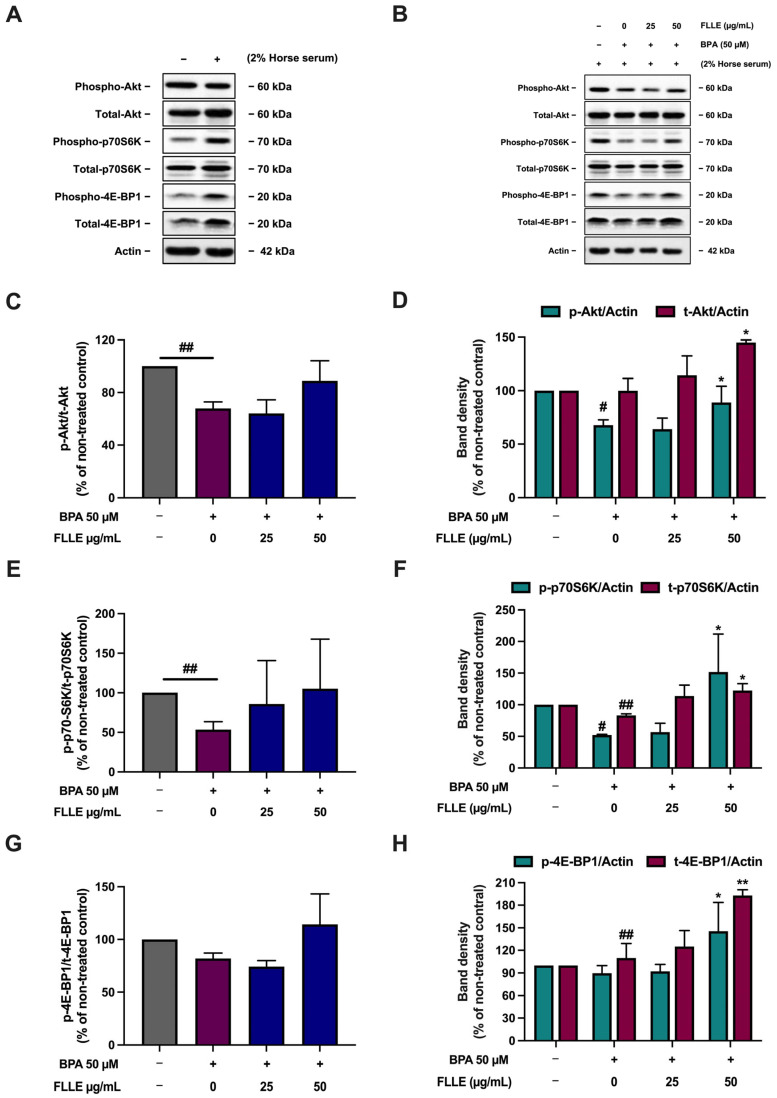
Effects of FLLE on the Akt/p70S6K/4EBP1 pathway in C2C12 myotubes. After the treatment, the protein samples were collected and used to determine Akt/p70S6K/4EBP1 phosphorylation levels via Western blotting. The relative phosphorylation levels of Akt, p70S6K, and 4EBP1 were calculated by dividing the amount of protein detected by the phosphorylated antibody by the amount detected by the antibody to determine total protein levels. (**A**) The level of the phospho- and total forms of Akt, p70S6K, and 4EBP1 in non-differentiated myoblasts compared to non-treated control myocytes. (**B**) A representative Western blotting result of three independent experiments. (**C**,**E**,**G**) Relative phosphorylation levels of Akt (**C**), p70S6K (**E**), and 4EBP1 (**G**). (**D**,**F**,**H**) Band density of phospho- and total forms of Akt (**D**), p70S6K (**F**), and 4EBP1 (**H**), normalized with β-actin levels. Each value in (**C**–**H**) represents mean ± SD (n = 3) * *p* < 0.05, ** *p* < 0.01 vs. BPA-treated control, ^#^ *p* < 0.05, ^##^ *p* < 0.01 vs. non-treated control.

The Akt and mTOR signaling pathway are required for skeletal muscle development [60], while BPA suppresses the activation of this pathway, leading to the inhibition of myogenesis [29]. To determine whether FLLE modulates the activation of the Akt/mTOR pathway in BPA-treated C2C12 cells, the phosphorylation of Akt, p70S6K, and 4E-BP1 was measured. Treatment of the cells with BPA significantly decreased the phosphorylation of Akt and p70S6K, but not 4E-BP1 phosphorylation (Figure 6). FLLE treatment slightly but not significantly increased the relative phosphorylation level of Akt and its key downstream targets, p70S6K and 4E-BP1, in the BPA-treated cells. The phosphorylation and total protein levels (normalized with β-actin) of Akt, p70S6K, and 4E-BP1 were significantly stimulated by FLLE (50 µg/mL) in the BPA-treated C2C12. This could suggest that FLLE (50 µg/mL) indirectly induces an Akt/mTOR signaling cascade through activating the expression of total Akt, p70S6K, and 4E-BP1 (Figure 6), resulting in an increase in the phosphorylation of these proteins.

These results are comparable with a previous study that showed that BPA significantly decreased the phosphorylation of Akt, p70S6K, and 4E-BP1 [29]. However, the dose and exposure time of BPA were different. In the previous study, the cells were treated with 1 µM BPA for 3 days of the differentiation process, whereas, in this study, the cells were treated with 50 µM BPA for 6 days of the differentiation process [29]. These differences may influence the effect of BPA on the Akt/p70S6K/4EBP pathway. A time-dependent BPA treatment could be a topic for further study.

## 3. Discussion

The reduction in or loss of skeletal muscle mass and diminished contractile strength are common and debilitating features of various human diseases, such as neuromuscular disorders, cancer, AIDS, and diabetes [61,62]. Previous research has also indicated that the deterioration of muscle function is linked to factors such as aging, a sedentary lifestyle, and lack of mobility. Additionally, the wasting of skeletal muscle due to aging and disease has been associated with elevated levels of inflammation [63]. Finding promising compounds that could promote muscle mass and functions can be a strategy to delay aging and improve the quality of human life.

C2C12 cells are mouse myoblasts originating from satellite cells, which have the ability to spontaneously differentiate into myotubes when moving from a high-serum to a low-serum culture medium. These cells serve as a valuable model for studying myoblast development, protein expression, and various molecular mechanisms [63]. In the present study, C2C12 cells were used as an in vitro model to examine whether *Ficus lindsayana* leaf extract (FLLE) protects C2C12 mouse myoblasts against the suppressive effects of bisphenol-A (BPA) on myogenic differentiation.

BPA and its metabolites are well-known obesogens that induce adipogenesis [22], modulate myogenic differentiation, and interact with endogenous estrogens. BPA has been proven to bind to hormone receptors, influencing several endocrine pathways [64,65,66]. Skeletal muscle is considered to be a target tissue for estrogens because it expresses two ER isoforms [22]. BPA contains two phenolic groups, allowing it to mimic estrogenic effects. This structural similarity is thought to interfere with myogenesis due to its estrogen-like chemical structure and potential to downregulate crucial myogenic regulatory proteins [67]. Moreover, BPA has been reported to cause cell cycle arrest and apoptosis, modify critical signaling pathways, and enhance oxidative stress in several types of normal cells [3,67,68]. These processes all contribute to its indicated negative effects on the proliferation and differentiation of muscle cells.

BPA, for instance, decreases Akt phosphorylation in skeletal muscle and affects serum adipocytokine levels, resulting in glucose intolerance [69]. In this study, the effects of BPA on myoblast differentiation were investigated and it was found that BPA disturbed the myogenic differentiation of C2C12 myoblasts. The finding was consistent with a previous report that showed that BPA inhibits myoblast differentiation and reduces myotube formation [29]. Moreover, BPA (50 µM) significantly decreased the cell viability of C2C12 myoblasts in the non-differentiated condition, suggesting the inhibition of cell proliferation. This result is comparable to a previous study that showed that BPA inhibits the division of C2C12 myoblasts [3]. The viability of BPA-stimulated C2C12 myoblasts increased significantly when co-treated with FLLE (200 µg/mL), suggesting that the extract may lessen the inhibitory effect of BPA on cell division. Our findings indicated that FLLE significantly enhanced the formation of neo-myotubes compared to the BPA-treated control cells, without affecting cell viability. The results demonstrated that FLLE promoted the fusion of myoblasts into multinucleated myotubes, as assessed through cell morphology analysis and staining with Jenner’s and Giemsa methods. Consequently, based on these results, we anticipated that FLLE would further stimulate myogenic differentiation in myoblasts exposed to BPA.

BPA exposure reduced Akt phosphorylation, which plays a critical role in myoblast differentiation through the promotion the expression of myogenic differentiation-related genes [29,63]. The treatment of the cells with BPA altered the phosphorylation of Akt and p70S6K but not 4E-BP. The dose and exposure time of BPA in this study were different to those used in a previous study, which used 1 µM BPA treatment for 3 days of the differentiation process, which significantly decreased the phosphorylation of Akt, p70S6K, and 4E-BP1 [29]. In this study, the cells were treated with 50 µM BPA for 6 days of the differentiation process. These differences may influence the effect of BPA on the Akt/4EBP/p70S6K pathway. A time-dependent treatment of BPA may be required in further studies. The treatment of 50 µg/mL FLLE led to an indirect increase in Akt phosphorylation in C2C12 cells exposed to BPA. In addition to enhancing Akt phosphorylation, this concentration of ELLE also triggered the phosphorylation of p70S6K and 4E-BP1, which are important downstream targets in the Akt/mTOR signaling pathway. These findings indicate that FLLE may counteract the reduction in myogenic differentiation caused by BPA by modulating the signaling pathways associated with myocyte differentiation. Nevertheless, expanding the scope of investigation to include other molecular markers of myogenesis might strengthen the findings and provide a more comprehensive understanding of the biological mechanisms underlying the impact of FLLE on the attenuation of BPA-suppressed myogenic differentiation.

In this study, the results of the HPLC analysis were consistent with our previous study, which reported that chlorogenic acid (CGA) could be found in the FL latex and root extracts and may act as an anti-inflammatory compound [59]. Catechin, rutin, and vanillic acid also were detectable in FLLE. Biological properties of chlorogenic acid, catechin, vanillic acid, and rutin have been reported, including antioxidant and anti-inflammation properties, anti-insulin resistance, and anti-obesity effects [70,71,72,73,74]; these compounds, which are contained in FLLE, could exert various bioactivities that should be further explored. Interestingly, previous studies reported on the protective ability of chlorogenic acid and catechin against muscle atrophy induced by various pro-atrophic factors, including toxic chemicals, and oxidative stress [75,76]. Furthermore, the effects of vanillic acid and rutin on the promotion of muscle health have been explored [77,78]. These data indicate that the phenolic and flavonoid compounds present in FLLE may significantly contribute to counteracting the inhibitory effects of BPA on myocyte differentiation.

At the molecular level, bisphenol A (BPA) induces significant damage through the increased generation of reactive oxygen species (ROS), disruption of the redox balance, mitochondrial dysfunction, and alterations in cell signaling pathways. To mitigate the effects of BPA, researchers are exploring the use of antioxidants. Studies have shown that both natural and synthetic antioxidants can help alleviate BPA’s toxicity and its harmful effects [41,79]. Compounds such as catechin, chlorogenic acid (CGA), rutin, and vanillic acid—which are found in *Ficus lindsayana* leaf extract (FLLE)—are recognized for their antioxidant and anti-inflammatory properties. Research indicates that green tea extract and epigallocatechin gallate (EGCG), a primary catechin in green tea, effectively prevent or reduce metabolic disorders and vascular toxicity linked to BPA due to their antioxidant and anti-inflammatory activities [80,81]. CGA has also demonstrated protective effects against BPA-induced toxicity, enhancing cell viability and reducing oxidative damage [82]. Additionally, CGA can safeguard against various chemical-induced toxicities, including those from fungal and bacterial toxins, pharmaceuticals, metals, and pesticides, by preserving cell survival through the reduction in excessive nitric oxide and ROS production while inhibiting pro-apoptotic signaling [82,83,84]. Rutin has been shown to counteract oxidative stress caused by BPA exposure. In studies with rats, rutin administration restored the biochemical parameters altered by BPA, such as liver enzyme levels and markers of oxidative stress. It also inhibits the generation of pro-inflammatory mediators through the MAPK and NF-kB pathways, suggesting its potential in reducing inflammation related to BPA toxicity [85,86]. Vanillic acid (VA) has been investigated for its protective effects against bisphenol S (BPS)—a derivative of BPA—and diethyl phthalate (DEP). VA has been found to alleviate the oxidative stress and tissue damage caused by these compounds, particularly in kidney and cardiac tissues [87]. In addition, recent research highlights the roles of bioactive compounds such as catechin, chlorogenic acid, rutin, and vanillic acid in promoting myoblast differentiation. Each of these compounds influences this process through distinct biochemical pathways, presenting opportunities for their use as functional foods to enhance muscle health. Catechin, CGA, rutin, and VA demonstrated potent effects on myoblast differentiation. They enhance the expression of key myogenic regulatory factors (MRFs) such as MyoD, MEF2A, and myogenin and promote changes in cell morphology that are indicative of successful differentiation. By enhancing mitochondrial function and reducing oxidative stress, they create an optimal environment for muscle cell maturation and growth and inhibit pathways associated with muscle degradation. In addition, the compounds have been shown to activate the Akt/mTOR signaling pathway, which is crucial for muscle cell growth and differentiation [77,78,88,89,90,91,92]. The collective evidence suggests that catechin, chlorogenic acid, rutin, and vanillic acid are promising candidate bioactive compounds in FLLE. Functional foods incorporating these compounds or FLLE could suppress BPA’s adverse effects and enhance myoblast differentiation more effectively than single-component supplements.

In conclusion, the findings of our study indicate that FLLE diminishes BPA-induced declines in skeletal muscle myoblast differentiation. This suggests that Akt-dependent regulation by FLLE may control the function of the myocyte differentiation-related signaling pathway. The research findings from this study provide valuable evidence of the health benefits of this plant, paving the way for its potential development as an alternative medicine, functional ingredient, or dietary supplement to alleviate health problems caused by BPA and other pollutants. Nonetheless, future studies should concentrate on identifying the key bioactive components and undertaking in vivo safety assessments of *Ficus lindsayana* extract (FLLE).

## 4. Materials and Methods

All methods were performed in accordance with the relevant guidelines and regulations.

### 4.1. Chemicals and Reagents

Bisphenol A (BPA) was obtained from Sigma (Darmstadt, Germany). Horse serum was purchased from Biochrom Ltd. (Cambridge, UK), and fetal bovine serum was purchased from HyClone (Pasching, Austria). Giemsa stain and Jenner’s (Leishman’ stain) were purchased from Alfa Aesar (Heysham, UK) and VMR International Ltd. (Spode, UK), respectively.

### 4.2. Plant Sample and Extraction

*Ficus lindsayana* (FLL) leaf samples were collected and deposited at the Thailand Natural History Museum (THNHM), Thailand Science Park, and assigned the code Chantarasuwan 040117-1. The extraction process involved soaking the dried leaves in 80% ethanol at a weight-to-volume ratio of 10:100 overnight, with occasional stirring; this procedure was repeated twice. The resulting ethanolic extract was concentrated using a rotary evaporator and subsequently freeze-dried with a lyophilizer. The yield of the ethanol extract (FLLE) was approximately 11.35%. The powder was then stored at −20 °C until use.

### 4.3. Phytochemical Analysis

A phytochemical analysis of FLLE was conducted using high-performance liquid chromatography (HPLC) with an Agilent 1260 Infinity II system (Agilent Technologies, Inc., Santa Clara, CA, USA), employing an Agilent ZORBAX Eclipse Plus (Agilent Technologies, Inc., Santa Clara, CA, USA) C18 column (250 × 4.6 mm, 5 μm). The chromatographic profile of the phytochemicals was established through a gradient method utilizing mobile phase A (1% acetic acid in water) and mobile phase B (100% acetonitrile), with a total runtime of 50 min at a flow rate of 0.7 mL/min. The gradient composition started with 90% A at 0 min, decreasing to 60% A by 28 min, then to 40% A for the next 39 min, and finally to 10% A by the end of the run. A sample of 20 mg/mL dissolved in 1 mL of methanol was injected into the column and detection occurred at 280 nm. HPLC data were recorded using a photodiode array detector and analyzed in triplicate with OpenLAB CDS ChemStation Edition Software 3.5. The retention times for the extract samples were compared against standard compounds such as gallic acid, chlorogenic acid, catechin, mangiferin, vanillic acid, caffeic acid, rutin, ferulic acid, rosmarinic acid, quercetin, apigenin, and kaempferol to identify the components present in the extract. The HPLC fingerprint of all standards used in this study is shown in Supplementary Figure S1A,B. The standard curve of the chlorogenic acid, rutin, vanillic acid, and catechin detectable in FLLE is shown in the Supplementary Data, Figure S1C–F.

### 4.4. Cell Culture and Treatment

A C2C12 myoblast cell line, acquired from the American Type Culture Collection (ATCC), was cultured in Dulbecco’s Modified Eagle Medium (DMEM) containing 4.5 g/L glucose, supplemented with L-glutamine, 10% fetal bovine serum (FBS), and 1% penicillin/streptomycin solution. The cells were maintained at 37 °C in a humidified incubator with 5% CO_2_. C2C12 cells were seeded in either 24- or 6-well plates and allowed to grow until they reached confluence, which took about 48 h (day 2). Following this, the cells were induced to differentiate using DMEM (high-glucose) supplemented with 2% horse serum as the differentiation medium. The fusion and differentiation processes continued for six days, with the conditioned medium being refreshed each day throughout this period [93].

To evaluate the effect of FLLE on the viability of C2C12 myoblasts, the cells were treated with various concentrations of the extract (0–800 µg/mL) for 48 h. The range of the FLLE concentration used in this experiment was estimated from our previous study, which found that the latex (LE) and root (RE) extract (0–800 μg/mL) of FL showed variations in terms of their cytotoxicity on various normal cell types, including mouse macrophages and human peripheral blood mononuclear cells [59].

To investigate the effect of BPA and FLLE co-treatment on the viability of C2C12 myoblasts under non-differentiated and differentiated conditions, the cells were treated with BPA (0, 50 μM) with or without FLLE (0–200 µg/mL) in either non-differentiated myoblasts for 3 days (72 h) or in cells undergoing differentiation for 6 days. At the indicated time, the cell viability was determined via MTT assay.

To determine the effect of FLLE on the myogenesis of BPA-treated C2C12 myoblasts, the cells were treated with the extracts (0–50 µg/mL) in the presence or absence of 50 µM BPA during differentiation for 6 days. The condition medium was changed each day. Then, the treated cells were subjected to further measurement.

### 4.5. Cytotoxicity Testing

Cytotoxicity was measured via 3-(4,5-Dimethylthiazol-2-yl)-2,5 Diphenyltetrazolium Bromide (MTT) assay, as described previously [94]. Briefly, at the indicated time, 0.5 mg/mL MTT solution was added to the treated cells and further incubated at 37 °C for 4 h. Following this incubation, the supernatant was removed and replaced with dimethyl sulfoxide (DMSO). The absorbance of formazan was measured with a microplate reader (Synergy HT, Hampton, NH, USA; BioTek, Rancho Cordova, CA, USA) at wavelengths of 540/630 nm. The percentage of cell survival was calculated using the following formula:

% cell survival = Abs_treatment/Abs_control × 100

### 4.6. Measurement of Fusion Index

An analysis of the C2C12 myotube fusion index was performed as previously reported [93,95] to determine myotube differentiation. After the treatment, the cells were washed twice with phosphate-buffer saline (PBS) and fixed with absolute methanol for 10 min. After that, the myotubes were stained with Jenner’s staining (1:3 *v*/*v* in 1 mM sodium phosphate solution for 10 min), followed by Giemsa staining (1:20 *v*/*v* in 1 mM sodium phosphate solution) for 10 min. The stained cells were observed under an optical microscope (ZEISS, Oberkochen, Germany; Axio Observation 7, Essen, Germany). Nine pictures were randomly taken from each well of the 24-well plates under each condition [95]. The diameters of three different sites in each myotube were measured using ImageJ 1.5g software, and at least 40 myotubes per condition were measured [93]. The fusion index was calculated as the number of nuclei in myotubes with three or more nuclei divided by the total number of nuclei present in a field of view.

### 4.7. Western Blot Analysis

Following treatment, the cells were harvested to assess the levels of differentiation of the signaling proteins. The cells were lysed to extract protein samples, which were then analyzed using either 10% or 12% SDS-PAGE. The proteins were subsequently transferred to a nitrocellulose membrane via an electric current. Specific antibodies were used to probe the target proteins, including anti-phospho-Akt (Ser473) at a dilution of 1:1000 (Cell Signaling, Danvers, MA, USA), anti-Akt at 1:2000 (Cell Signaling, USA), anti-phospho-p70 S6 Kinase (Thr389) at 1:1000 (Cell Signaling, USA), and anti-p70 S6 Kinase at 1:2000 (Cell Signaling, USA). Additional antibodies included anti-phospho-4E-BP1 (Thr37/46) at 1:1000 (Cell Signaling, USA), anti-4E-BP1 at 1:1000 (Cell Signaling, USA), anti-myogenin at 1:1000 (Santa Cruz, CA, USA), anti-MEF2A at 1:1000 (Santa Cruz, CA, USA), and anti-β-actin at 1:10,000 (Sigma, Burbank, CA, USA). The resulting visible bands appeared at molecular weights of 60 kDa for phospho-Akt and total Akt, 70 kDa for phospho-p70 S6 Kinase and total p70 S6 Kinase, 20 kDa for phospho-4E-BP1 and total 4E-BP1, 34 kDa for myogenin, 54 kDa for MEF2A, and 42 kDa for β-actin. The Western blot density of each protein band was quantified using the ImageJ software, NIH, Bethesda, MD, USA.

### 4.8. Statistical Analysis

All values are presented as mean ± standard deviation ($\bar{X}$ ± SD) based on triplicate samples from three independent experiments. The overall differences between treatment groups were assessed using a one-way analysis of variance (ANOVA) followed by post hoc Tukey’s test, conducted with Prism version 10 (GraphPad Software, Boston, MA, USA), or through Student’s *t*-test. A *p*-value of less than 0.05 is considered statistically significant.

## Figures and Tables

**Table 1:** The quantitative phytochemical compounds in FLLE extracts obtained using HPLC.

	mg/g Extract			
	Chlorogenic Acid	Vanillic Acid	Rutin	Catechin
FLLE	3.09 ± 0.076	0.97 ± 0.020	5.51 ± 0.155	1.08 ± 0.053

The results are expressed as mean ± SD, n = 3.

## Data Availability

The datasets used and/or analyzed during the current study are available from the corresponding author upon reasonable request.

## Supplementary Materials

The following supporting information can be downloaded at: https://www.mdpi.com/article/10.3390/ijms26020476/s1.
