# Supplementary material for: Ficus lindsayana Leaf Extract Protects C2C12 Mouse Myoblasts Against the Suppressive Effects of Bisphenol-A on Myogenic Differentiation
Source: Int J Mol Sci. 2025 Jan 8;26(2):476. doi: 10.3390/ijms26020476 (PMC11765284; doi:10.3390/ijms26020476)

Ficus lindsayana Leaf Extract Protects C2C12 Mouse Myoblasts Against the Suppressive Effects of Bisphenol-A on Myogenic Differentiation

Pornsiri Pitchakarn [1], Jirarat Karinchai [1], Pensiri Buacheen [1], Arisa Imsumran [1], Ariyaphong Wongnoppavich [1], Kongsak Boonyapranai [2] and Sakaewan Ounjaijean [2,3,*]

1 Department of Biochemistry, Faculty of Medicine, Chiang Mai University, Chiang Mai 50200, Thailand; pornsiri.p@cmu.ac.th (P.P.); jirarat.ka@cmu.ac.th (J.K.); pensiri.bua@cmu.ac.th (P.B.); arisa.bonness@cmu.ac.th (A.I.); ariyaphong.w@cmu.ac.th (A.W.)

2 Research Center for Non-Infectious Diseases and Environmental Health Sciences, Research Institute for Health Sciences, Chiang Mai University, Chiang Mai 50200, Thailand; kongsak.b@cmu.ac.th

3 School of Health Sciences Research, Research Institute for Health Sciences, Chiang Mai University, Chiang Mai 50200, Thailand

* Correspondence: sakaewan.o@cmu.ac.th

Supplementary Figure S1. HPLC fingerprint of known standard compounds used in this study (S1A-B) and the standard curve of chlorogenic acid, rutin, vanillic acid and catechin (S1-C-F). The phytochemical analysis of FLLE was conducted using high-performance liquid chromatography (HPLC) with an Agilent 1260 Infinity II system, employing an Agilent ZORBAX Eclipse Plus C18 column (250 × 4.6 mm, 5 µm). The chromatographic profile of the phytochemicals was established through a gradient method utilizing mobile phase A (1% acetic acid in water) and mobile phase B (100% acetonitrile), with a total runtime of 50 minutes at a flow rate of 0.7 mL/min. The gradient composition started with 90% A at 0 minutes, decreasing to 60% A by 28 minutes, then to 40% A for the next 39 minutes, and finally to 10% A by the end of the run. A sample of 20 mg/mL dissolved in 1 mL of methanol was injected into the column, and detection occurred at 280 nm. HPLC data were recorded using a photodiode array detector and analyzed in triplicate with OpenLAB CDS ChemStation Edition Software 3.5. The retention times for the extract samples were compared against standard compounds such as gallic acid, chlorogenic acid, catechin, mangiferin, vanillic acid, caffeic acid, rutin, ferulic acid, rosmarinic acid, quercetin, apigenin, and kaempferol to identify the components present in the extract. (A) HPLC chromatogram of Catechin (A), Caffeic acid (B), Rutin (C), Rosmarinic acid (D), Quercetin (E), Apigenin (F), Kaempferol (G). (B) HPLC chromatogram of Gallic acid (A), Chlorogenic acid (B), Mangiferin (C), Vanillic acid (D), Ferulic acid (E).

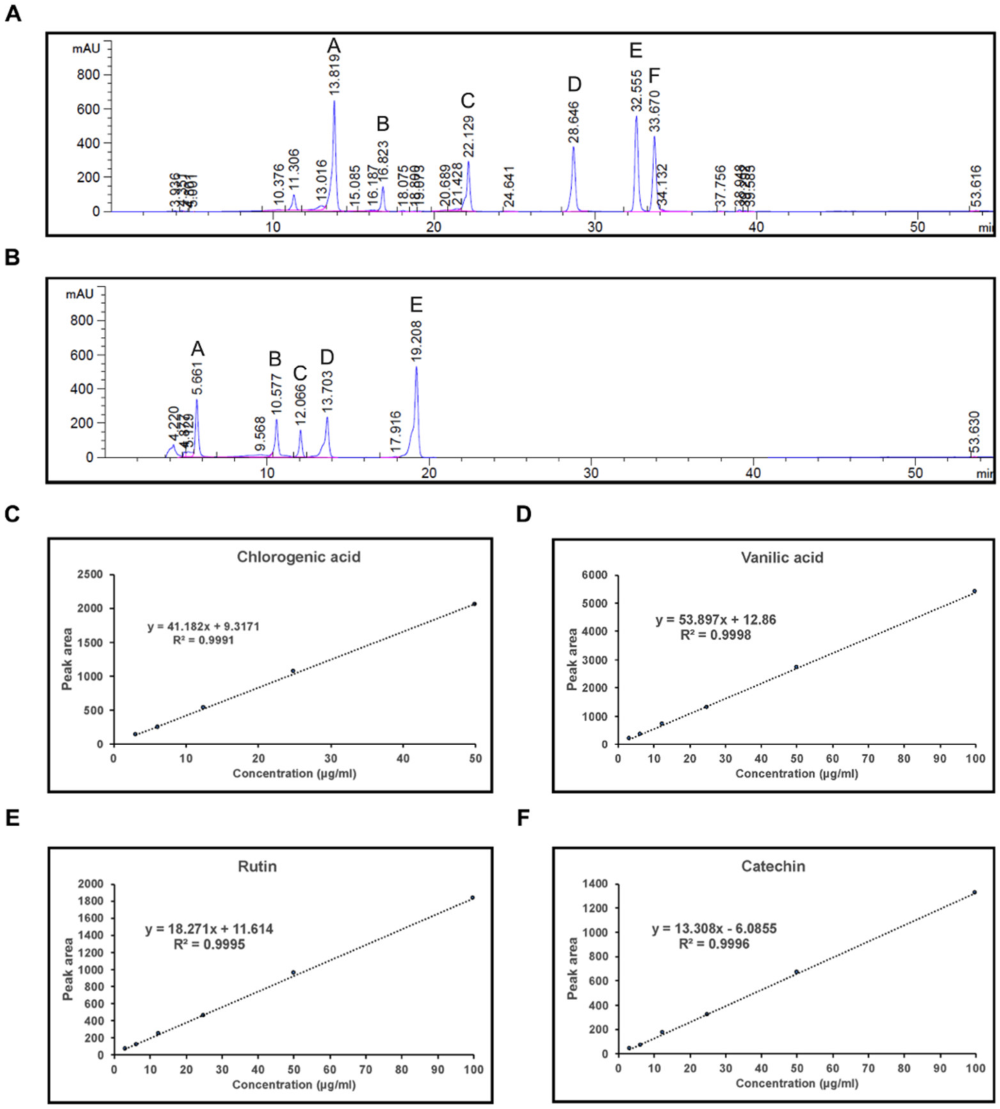

Supplementary Figure S2. Myogenesis inhibitory effect of BPA in C2C12 cells. To determine the effect of FLLE on myogenesis of BPA-treated C2C12 myoblasts. The cells were treated with the 25 or 50 µM of BPA during differentiation for six days. Then the treated cells were subjected for the measurement of fusion index. * $p < 0.05$ vs. non-treated control.

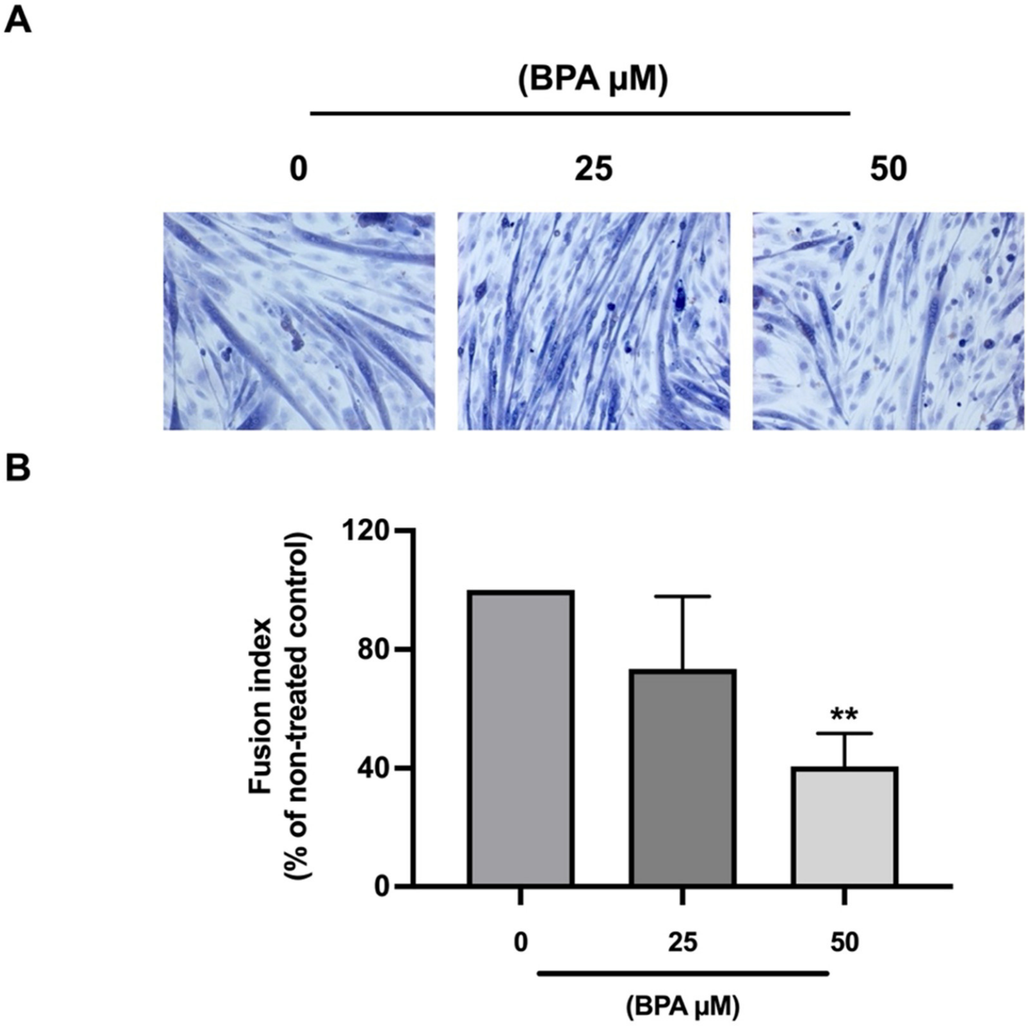

Supplementary Figure S3. Band density of Western blot results from tree-time independent experiments measuring the effect of FLLE on myogenin protein level in the BPA-exposed cells. To determine the effect of FLLE on myogenesis of BPA-treated C2C12 myoblasts. The cells were treated with the extracts (0-50 µg/ml) in the presence or absence of 50 µM BPA during differentiation for 6 days. Following treatment, the cells were harvested to assess the level of myogenin by Western blotting.

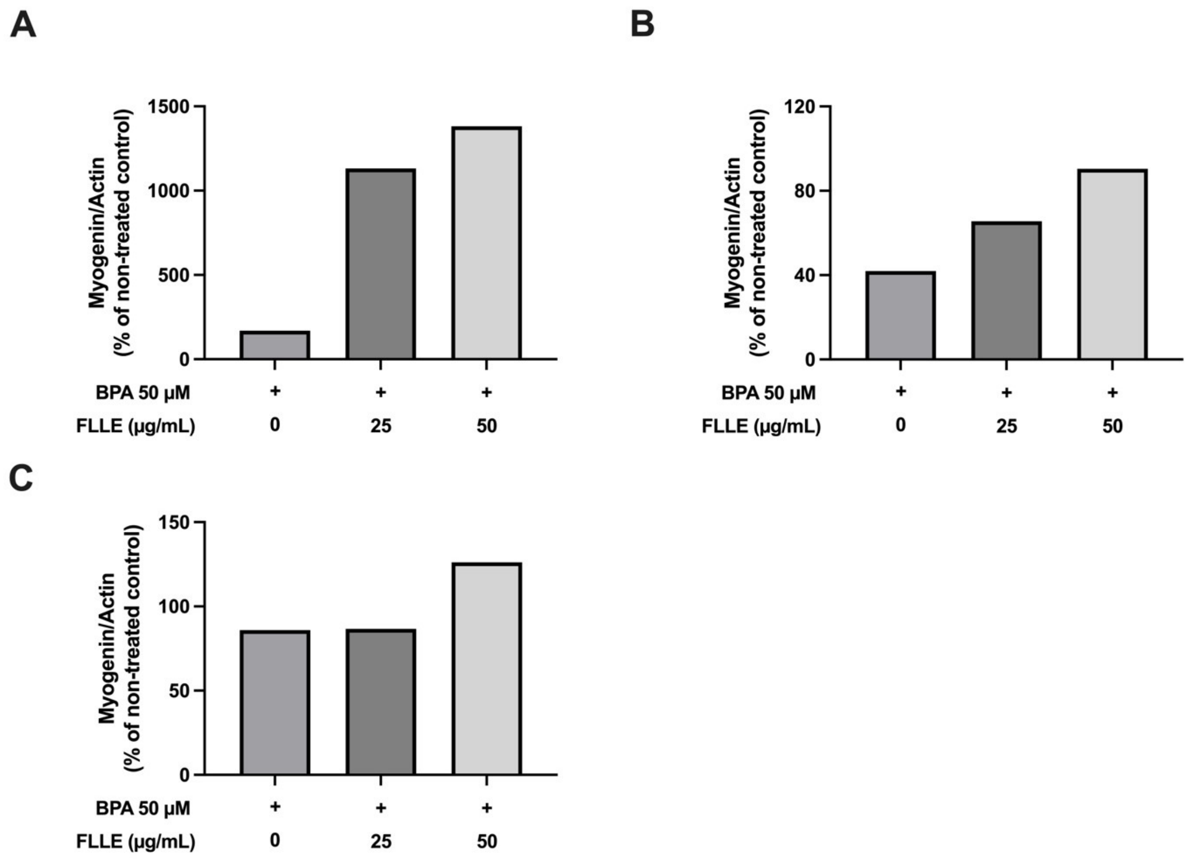

Supplement: Supplementary file 1 [file ijms-26-00476-s001.zip › ijms-3165221-supplementary.pdf]
